# Elder (*Sambucus nigra*), identified by high-content screening, counteracts foam cell formation without promoting hepatic lipogenesis

**DOI:** 10.1038/s41598-024-54108-7

**Published:** 2024-02-12

**Authors:** Stefanie Steinbauer, Alice König, Cathrina Neuhauser, Bettina Schwarzinger, Herbert Stangl, Marcus Iken, Julian Weghuber, Clemens Röhrl

**Affiliations:** 1 University of Applied Sciences Upper Austria, Stelzhamerstrasse 23, 4600 Wels, Austria; 2 Austrian Competence Center for Feed and Food Quality, Safety and Innovation, Wels, Austria; 3 Center for Pathobiochemistry and Genetics, Institute of Medical Chemistry, Medical University of Vienna, Vienna, Austria; 4 PM International AG, Schengen, Luxembourg

**Keywords:** Lipoproteins, Cardiovascular diseases

## Abstract

Cholesterol deposition in intimal macrophages leads to foam cell formation and atherosclerosis. Reverse cholesterol transport (RCT), initiated by efflux of excess cholesterol from foam cells, counteracts atherosclerosis. However, targeting RCT by enhancing cholesterol efflux was so far accompanied by adverse hepatic lipogenesis. Here, we aimed to identify novel natural enhancers of macrophage cholesterol efflux suitable for the prevention of atherosclerosis. Plant extracts of an open-access library were screened for their capacity to increase cholesterol efflux in RAW264.7 macrophages trace-labeled with fluorescent BODIPY-cholesterol. Incremental functional validation of hits yielded two final extracts, elder (*Sambucus nigra*) and bitter orange (*Citrus aurantium L*.) that induced ATP binding cassette transporter A1 (ABCA1) expression and reduced cholesteryl ester accumulation in aggregated LDL-induced foam cells. Aqueous elder extracts were subsequently prepared in-house and both, flower and leaf extracts increased ABCA1 mRNA and protein expression in human THP-1 macrophages, while lipogenic gene expression in hepatocyte-derived cells was not induced. Chlorogenic acid isomers and the quercetin glycoside rutin were identified as the main polyphenols in elder extracts with putative biological action. In summary, elder flower and leaf extracts increase macrophage ABCA1 expression and reduce foam cell formation without adversely affecting hepatic lipogenesis.

## Introduction

Despite great advances in cholesterol-lowering pharmacological therapy extending life span, many individuals with cardiovascular events history suffer from poor quality of life often associated with consequences of atherosclerosis. Therapeutic interventions managed to lower LDL cholesterol levels, but interventions on other areas, including HDL and foam cells, are yet unfinished^1,2^. In addition, pharmacological treatment often faces the issue of nonadherence of patients to lipid-lowering therapy, frequently linked to drug-associated side-effects^3^. Natural products that affect cholesterol metabolism are promising in preventing atherosclerosis, for treating persons with borderline lipid profiles and are effective on top of pharmacological drugs^4^.

Atherosclerosis, the deposition of fatty and/or fibrous material in the intima of arteries, is the primary root for vascular diseases^3^. A main contributor to cholesterol deposition in the arteries is the accumulation of cholesterol in macrophages^1^. Lipid-laden macrophages—termed foam cells—are already found in early atherosclerotic lesions termed “fatty streaks” and are involved in the progression of the disease as they lack negative-feedback regulation of cholesterol uptake. This leads to cellular lipid-overload that eventually results in apoptotic cell death accompanied by the release of atherogenic and inflammatory components into the atheroma promoting both plaque growth and rupture^5^.

A therapeutic approach to reduce intimal cholesterol deposition is to increase reverse cholesterol transport (RCT) from macrophage foam cells to the liver for excretion of cholesterol via bile and feces, thereby preventing or even regressing atherogenesis^6^. In this regard, macrophage cholesterol efflux to apolipoprotein (apo)A–I and HDL molecules is the first step of the atheroprotective RCT. Indeed, high cholesterol efflux capacity of human sera—a higher ability of HDL subclasses to accept cholesterol from macrophages—correlates with reduced intima media thickness and reduced incidence of cardiovascular events^7,8^.

On the molecular level, macrophage cholesterol efflux is initiated by delivery of cholesterol onto apoA-I and HDL particles via ATP-binding cassette (ABC) transporters ABCA1, ABCG1 and the scavenger receptor class B, type 1 (SR-BI). While ABCA1 transfers cholesterol onto lipid poor HDL (also termed discoidal or nascent HDL), ABCG1 and SR-BI prefer lipid rich HDL (also termed spherical or mature HDL). In macrophage foam cells, ABCA1 is regarded the quantitatively most important transporter for cholesterol efflux^9^.

We aimed to identify novel plant extracts that increase macrophage cholesterol efflux and reduce foam cell formation. Therefore, a high-content screen was developed and applied to aqueous plant extracts from the open-source library PECKISH^10^ containing a broad diversity of extracts regarding botanical families, origin and plant parts. Top hits of the screening assay were thoroughly validated in human and murine macrophage models in terms of their ability to reduce foam cell formation and to alter expression of relevant cholesterol efflux transporters.

## Methods

### Plant extracts

642 aqueous plant extracts of the open-access plant extract collection Kiel in Schleswig–Holstein (PECKISH^10^) were tested in the screening approach at 1/1000 dilution (≙ 5–10 µg/mL).

For in-depth investigation of elder effects, aqueous extracts were prepared in-house from elder flowers, leaves or berries collected in the district of Upper Austria (Austria, Europe). For cold extraction, plant parts were dried (50 °C, 18 h) and ground; 5 g thereof were dissolved in 30 mL double-distilled water at room temperature, vortexed and sonicated for 30 min. After vortexing, samples were incubated in an overhead shaker at room temperature for 2 h and centrifuged (3157 × g, 10 min, RT). The supernatant was collected; the residue was rinsed with water, vortexed, centrifuged and both supernatants were combined. Final volume of plant extract was 50 mL. Aliquots of the extracts were stored at − 80 °C until further use. Hot extracts were prepared accordingly, except 70 °C hot water was used as solvent, no sonication was carried out and overhead shaking duration time was 10 min. Extract dry matter was determined using a MA160 Moisture Analyzer (Sartorius, Göttingen, Germany). For experiments, extracts were diluted to concentrations of up to 500 µg/mL dry matter, which did not reduce cell viability in macrophages as assessed by resazurin-assays (data not shown).

### Cell culture

Cell lines were cultivated under standard conditions (37 °C, 5% CO2, ≥ 95% humidity) and regularly tested for mycoplasma infections.

RAW264.7 murine macrophages (ATCC®: TIB-71™; City of Manassas, VA, USA) were cultivated in Dulbecco’s Modified Eagle’s Medium (DMEM; 4.5 g/L glucose) containing 10% fetal bovine serum (FBS) and 1% penicillin/streptomycin (P/S; final concentration: 100 U/mL penicillin and 0.1 mg/mL streptomycin; all from PAN-Biotech GmbH, Aidenbach, Germany). Cells were subcultured at 60–80% confluency by detachment using a cell scraper. Maximum passage number used was 30, as recommended previously^11^.

Human monocytic THP-1 cells (ATCC®: TIB-202™) were cultivated in RPMI 1640 with 2 mM L-glutamine, 1 mM sodium pyruvate, 10 mM HEPES, 4.5 g/L glucose, 1.5 g/L NaHCO_3_, 10% FBS, 1% P/S (PAN Biotech) and 50 µM sterile-filtered β-mercaptoethanol (Sigma-Aldrich, Saint Louis, MO, USA). For subculturing, cells were pelleted by centrifugation (120 × g, 7 min) and resuspended in fresh medium. For experiments, differentiation into macrophages was induced by the addition of 50 ng/mL phorbol 12-myristate 13-acetate (PMA; Sigma-Aldrich) for 48 h.

Human hepatic Huh-7 cells (ATCC®: JCRB-0403™) were cultivated in DMEM (4.5 g/L glucose) containing 10% FBS and 1% P/S (all from PAN Biotech) and subcultured at 80–90% confluency using standard trypsin detachment.

### Preparation of apoB-depleted serum

Polyethylene glycol (PEG) was used to precipitate apoB-containing lipoproteins^12^. Briefly, 40 parts PEG solution (20% PEG in 200 mM glycine buffer, sterile-filtered, pH = 7.4; Promega, Madison, USA) were added to 100 parts of sterile human serum from human male AB plasma (Sigma-Aldrich), vortexed and incubated at room temperature for 20 min. Then, apoB-containing precipitates were sedimented by centrifugation (10,000 × g, 4 °C, 30 min), the supernatant was collected and aliquots were stored at − 80 °C.

### Preparation of labeling medium

For labeling cells with the fluorescent cholesterol tracer BODIPY-cholesterol (TopFluor® Cholesterol; Avanti Polar Lipids, Alabaster, AL, USA), a previously published protocol^13^ was applied with slight modifications. 1.130 mL of cholesterol (Sigma-Aldrich) and 0.433 mL of BODIPY-cholesterol (both 1 mg/mL in Folch) were mixed in a round glass bottle and dried under nitrogen. The lipid film was resuspended in 15 mL of methyl-β-cyclodextrin (CD; Sigma-Aldrich; 20 mM in DMEM buffered with 10 mM HEPES). After sonification for 30 min, the sample was further incubated in a water bath at 37 °C for 3 h and agitated every 30 min. This pre-labeling medium was stored in the dark at 4 °C until further use. Calculated molar ratios of cholesterol/CD and BODIPY-cholesterol/unlabeled cholesterol were 1/80 and 1/4, respectively.

For cholesterol efflux experiments, labeling medium was freshly prepared from pre-labeling medium. For this purpose, pre-labeling medium was sonicated for 30 min, diluted 1/16 with DMEM containing 10 mM HEPES in a glass vial, filtered through a 0.45 µm filter and incubated at 37 °C for 1 h before use.

### Cholesterol efflux screening assay

**Figure 1:**
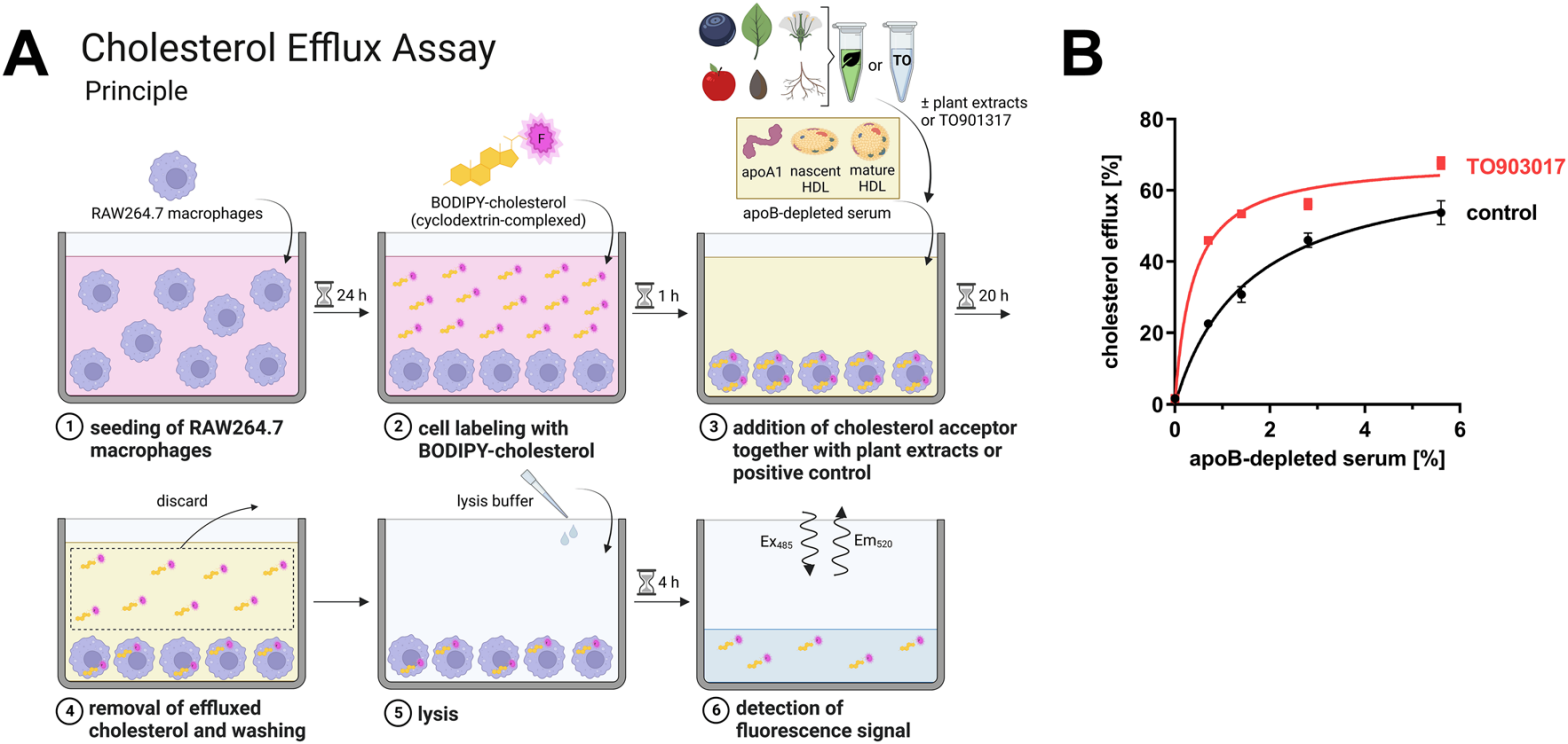
Screening assay principle and validation. (**A**) Overview of cholesterol efflux measurements, for details see Methods section. Created with BioRender.com. (**B**) Cholesterol efflux in RAW264.7 macrophages in dependence of acceptor concentration. BODIPY-cholesterol labeled cells were left untreated (control) or treated with TO901317 (10 µM) for 20 h, then apoB-depleted serum was added at the indicated concentrations for 4 h. Fluorescence signal of BODIPY-cholesterol in cell lysates was measured. Data of one experiment performed in triplicates are shown.

On day 0.5 × 10^4^ RAW264.7 cells/well (final volume: 200 µL/well) were seeded into black 96-well-plates with translucent bottom (Greiner Bio-One GmbH, Kremsmünster, Austria) (also see Fig. 1A). On day 1, cell medium was aspired and 100 µL of labeling medium were added under light protection followed by incubation at 37 °C for 1 h. Next, cells were washed with Hanks' Balanced Salt Solution (HBSS; PAN-Biotech GmbH) and treated with plant extracts of the PECKISH library (1/1000) or the LXR-agonist TO901317 (TO; 10 µM; Sigma-Aldrich) as positive control in DMEM containing 0.7% apoB-depleted serum in a final volume of 200 µL for 20 h. A negative control group was included where apoB-depleted serum was omitted. Afterwards, extracellular cholesterol was removed and cells were washed with ice-cold PBS. Next, 1% cholic acid (Sigma-Aldrich) dissolved in 0.17 M NaOH was added as lysis buffer. After 4 h lysis in the dark at room temperature, cellular BODIPY-cholesterol was measured fluorometrically (Ex: 485 nm, Em: 520 nm) using a POLARstar Omega microplate reader (BMG LABTECH, Ortenberg, Germany). Cholesterol efflux was calculated as the loss of intracellular labeled cholesterol. In detail, specific efflux to apoB-depleted serum was calculated by subtracting the values in cells treated with apoB-depleted serum from the values of the negative control group containing no acceptor. Data from screening were normalized to untreated controls containing only apoB-depleted serum.

### Gene expression analysis

On day 0.1 × 10^6^ (RAW264.7), 2.75 × 10^6^ (THP-1) or 1.8 × 10^5^ (Huh-7) cells were seeded into 6-well plates (Greiner Bio-One GmbH). On day 2, cells were washed once with PBS^+/+^ (containing Ca und Mg; PAN-Biotech GmbH), and plant extracts of the PECKISH library (1/1000), in-house extracts of elder (100, 200 and 500 µg/mL, cold and hot extracts) or TO (10 µM) were added in serum-reduced medium (1% FBS).

After 24 h incubation, RNA was isolated using the RNeasy® Plus Mini Kit from QIAGEN (Hilden, Germany). RNA-isolation was followed by cDNA-synthesis using the iScript™ cDNA Synthesis Kit from Bio-Rad Laboratories (Hercules, CA, US).

Reverse transcription qPCR (RT-qPCR) was carried out by a singleplex assay using iQ™ SYBR® Green Supermix (Bio-Rad Laboratories). Gene expression was analyzed according to standard protocols, primer sequences are found in Supplementary Table S1. Gene expression was normalized to average expression levels of two housekeeping genes (HPRT1 and GUSB for RAW264.7; RPL5 and GAPDH for THP-1 cells; B2M and GAPDH for Huh-7).

### Efflux of radiolabeled cholesterol

RAW264.7 cells (2 × 10^5^ cells/well) were seeded into 24-well plates on day 0 and trace labelled with 1 µCi/well ^14^C-cholesterol (Amersham Biosciences, Amersham, UK) in media containing 10% FBS on day 2. After 8 h, cells were washed with PBS and plant extracts of the PECKISH library (1/1000) or TO901317 (10 µM) were added in serum reduced media (1% FBS) for another 12 h. On day 3, cells were washed twice with PBS and incubated in DMEM containing 0.7% apoB depleted serum for 4 h. Supernatants were collected, centrifuged to remove cell debris, and analyzed by β-counting (Tri-Carb 2800 TR, Perkin Elmer). Cells were washed with PBS, lysed with 0.1 M NaOH and cell lysates were likewise analyzed by β-counting. Cholesterol efflux was calculated by dividing supernatant counts by total counts (i.e. supernatant plus cell lysate counts) and expressed in percent.

### Foam cell experiments

Aggregated LDL was prepared from human native LDL, that was isolated from human plasma of healthy normolipidemic volunteers via sequential flotation ultracentrifugation^14^. The use of plasma from human donors was approved by the ethics committee at the Medical University of Vienna, #1414/2016). Experiments were performed in accordance with the Helsinki Guidelines. Informed consent was obtained from all donors. Aggregation of LDL was accomplished by vigorous vortexing for 30 s. After centrifugation (18,000 × g, 4 °C, 10 min), the supernatant was removed and pelleted aggregated LDL was resuspended in DMEM containing 20 mM HEPES. Aggregated LDL was stored under nitrogen atmosphere in a glass vial at 4 °C until further use.

On day 0, RAW264.7 cells were seeded into 6 cm dishes (Greiner Bio-One GmbH) at a density of 3 × 10^6^ cells/dish. On day 2, cells were washed once with PBS^+/+^. Then cells were treated with 200 µg/mL agg-LDL in DMEM containing 10% FBS with or without addition of plant extracts from the PECKISH library (1/1,000) or TO as positive control (10 µM). After 24 h, cells were processed for gas chromatography.

### Gas chromatography

Cellular free and esterified cholesterol were measured by gas chromatography as previously described^15^. Briefly, cells were washed and detached by scraping in ice-cold PBS. After centrifugation (200 × g, 4 °C, 5 min) the pellet was resuspended in 1 mL distilled water. An aliquot of 60 µL was lysed with 240 µL NaOH (0.1 M) and subjected to cell protein determination via the Bradford assay for subsequent normalization. Remaining cells were extracted by standard Folch extraction. Lipids were quantitated using a chromatograph equipped with a programmed temperature vaporizer injector, a ZB-5HT capillary column (15 m × 0.32 mm × 0.1 μm; Phenomenex, Aschaffenburg, Germany) and an FID detector. Standards used for free cholesterol and cholesteryl ester were tridecanoyl glycerol and cholesteryl myristate (both from Sigma-Aldrich), respectively. LabSolutions (Shimadzu Corporation, Kyoto, Japan, vers. 5.84) was used for manual peak integration. Values were normalized to cell protein. Total cholesterol was calculated as the sum of free and esterified cholesterol.

### Western blot analysis

Cells were seeded and treated as described above for RT-qPCR. After 24 h, cells were washed with PBS and lysed with Cell Lysis Buffer (Cell Signaling Technology, Danvers, MA, USA) containing 1% Protease Inhibitor Cocktail (Sigma-Aldrich) on ice. After harvesting, sonication and centrifugation (14,000 × g, 10 min, 4 °C), supernatants were collected. Protein contents were quantified using the Micro BCA™ Protein Assay Kit from Thermo Fisher Scientific (Waltham, MA, USA).

Twenty µg of protein were separated by SDS–polyacrylamide electrophoresis under reducing conditions. Proteins were transferred onto a PVDF membrane (0.2 μm; Bio-Rad Laboratories) using the Trans-Blot Turbo Transfer System (protocol Bio-Rad Laboratories). Membranes were blocked with 1% blocking solution (Roche Diagnostics, Mannheim, Germany for 1 h). Primary antibodies against ABCA1, (1/500; Novus Biologicals, Centennial, CO, USA; #NB400-105), LXRα (1/500; Thermo Fisher; #PA1-330), β-actin (1/1000; Cell Signaling Technology; #4967) and GAPDH (1/100; Cell Signaling Technology; #5174) were diluted in 0.5% blocking solution and incubated at 4 °C overnight. Proteins were detected by standard protocols using horseradish peroxidase-conjugated secondary antibodies. Band intensities were analyzed semi-quantitatively using Image-Lab™ (Bio-Rad Laboratories) and protein expression was normalized to β-actin and relative to control.

### Analytics of polyphenols

Extract analyses were performed using reversed-phase chromatography, as described previously^16^ using a Ultimate 3000 HPLC system comprised of a LPG-3400SD pump with built-in degasser, a WPS-3000 U(T)SL cooled autosampler, a temperature-controlled column compartment and an DAD-3000(RS) diode array detector equipped with the Chromeleon software (all Thermo Scientific, Dreieich, Germany). Analyte separation was performed on an Accucore C18 column (150 mm × 3.0 mm × 2.6 μm; Thermo Scientific). Column temperature was 40 °C and the injection volume 1 μL. Detection wavelength was 260 nm. Analytes were separated via gradient elution with mobile phase A containing 0.1% formic acid (FA) in water and mobile phase B containing 0.1% FA in acetonitrile at a flow rate of 0.5 mL/min. The elution gradient starting conditions were 95% A and 5% B. After 5 min of equilibration time, the proportion of B was increased to 20% at 8 min and to 40% at 12 min, followed by 60% B at 15 min and 80% B at 17 min for 3 min. B was reduced to 5% at 20 min until 25 min.

Chlorogenic acid (3-CQA, Sigma Aldrich), neochlorogenic acid (5-CQA, Sigma Aldrich), rutin (Sigma-Aldrich) and isoquercetin (Extrasynthese, Genay, France) were used as standards. 4-CQA was assigned tentatively.

#### Statistical analysis

Data are shown as mean ± SD. Statistically significant differences were evaluated using Graph Pad Prism (Graph Pad Software, CA, USA; vers. 9.3.1). Data in Fig. 1B were fit by hyperbolic regression (Y = Bmax × X/(Kd + X)). For comparison of two groups, t-test with Welch’s correction was used. For comparison of more than two groups, ordinary ANOVA with Dunnett’s multiple comparisons test was performed. Significant p-values are marked by *(≤ 0.05), **(≤ 0.01), ***(≤ 0.001) or ****(≤ 0.0001).

## Results

### Screening approach

We aimed to identify natural extracts with anti-atherosclerotic effects beyond LDL-cholesterol lowering. Therefore, a screening assay to assess macrophage cholesterol efflux, a crucial step in reverse cholesterol transport, was established. RAW264.7 murine macrophages were trace-labeled with the cholesterol analog BODIPY-cholesterol (Fig. 1A). Cholesterol export was monitored after addition of human apoB-depleted serum which is devoid of the cholesterol donors VLDL and LDL while containing only HDL and subclasses thereof. To ensure experimental conditions under a dynamic range of cholesterol efflux, the dose-dependency of cholesterol efflux was observed under different acceptor concentrations. Figure 1B shows that cholesterol efflux was saturable at a concentration of ~ 5% apoB-depleted serum. Activation of cholesterol efflux transporter expression using the synthetic agonist LXR agonist TO901317 resulted in increased efflux yielding an ~ twofold increase of cholesterol efflux at a concentration of 0.7% apoB-depleted serum. Accordingly, this concentration was chosen for subsequent screening experiments.

**Figure 2:**
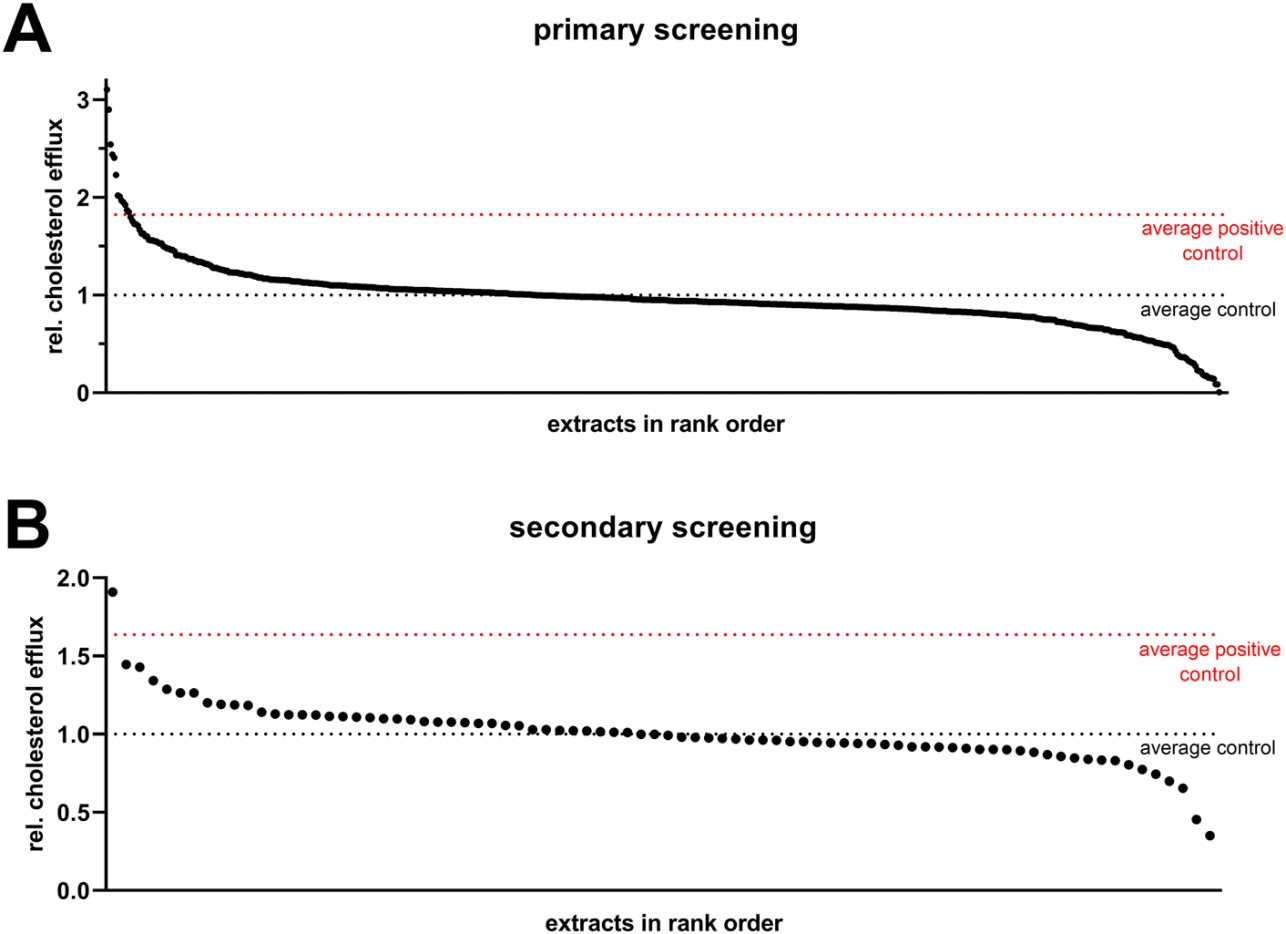
Screening of aqueous plant extracts on altering macrophage cholesterol efflux. Cholesterol efflux was measured using fluorescent BODIPY-cholesterol and apoB-depleted serum as acceptor. For details, see Methods section. Eighty-two hits from the first screening round (comprised of 642 extracts; **A**) were validated in a second screening round (**B**).

To this end, 642 aqueous plant extracts derived from the PECKISH library were subjected to the established screening assay (Fig. 2A). Data of 31 extracts were excluded due to apparent toxic effects (as judged by visual observation) or implausible results (possibly caused by plant extract autofluorescence) and were excluded from analyses. In the first screening round, 82 extracts were identified to increase cholesterol efflux, and 10 extracts thereof exerted stronger effects than TO901317, which served as a positive control (Fig. 2A). In a second screening round, 31 plant extracts were confirmed to augment cholesterol efflux (Fig. 2B).

### Validation

**Figure 3:**
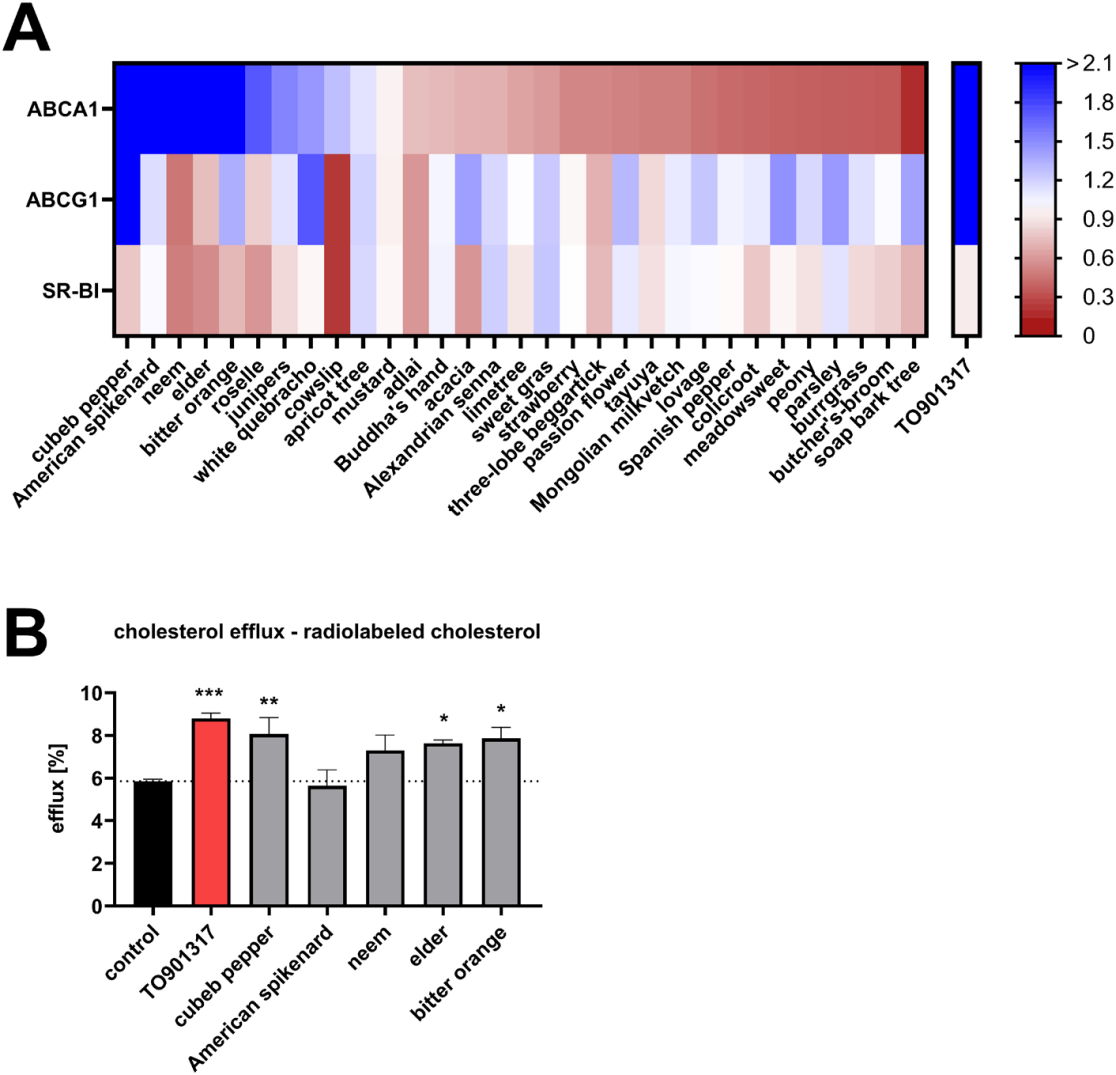
Validation of hits from screening via gene expression analysis of cholesterol efflux transporters. RAW264.7 mouse macrophages were treated with PECKISH extracts (5–10 µg/mL) or TO901317 (10 µM) for 24 h in serum-reduced media. Subsequently, RNA was extracted and analyzed by RT-qPCR (**A**). Mean values of relative mRNA expression of ABCA1, ABCG1 and SR-BI (n = 2) are shown. (**B**) Top hits were validated to enhance cholesterol efflux using radiolabeled cholesterol as described in the Methods section. Relative cholesterol efflux values are shown (n = 3).

Gene expression analysis by RT-qPCR was utilized for further characterization of identified inducers of cholesterol efflux. Beside aqueous diffusion, cholesterol is exported by ABCA1, ABCG1 and SR-BI, which are considered the most important efflux transporters counteracting macrophage foam cell formation^9^. Thus, expression levels of ABCA1 and ABCG1, which export excess cholesterol to nascent and mature HDL, respectively, as well as SR-BI, which mediates bidirectional cholesterol flux between HDL and cells, were analyzed in RAW264.7 cells. Seven plant extracts induced expression of ABCA1 > 1.5-fold, while only three extracts induced ABCG1 > 1.5-fold and none induced SR-BI expression > 1.5-fold (Fig. 3A).

To confirm the reliability of the applied screening assay, most effective extracts were subjected to analysis of cholesterol efflux as measured using radiolabeled cholesterol (Fig. 3B). Extracts of cubeb pepper, elder and bitter orange significantly induced cholesterol efflux. While neem extract displayed a trend towards increasing cholesterol efflux, an extract of American spikenard was ineffective.

**Figure 4:**
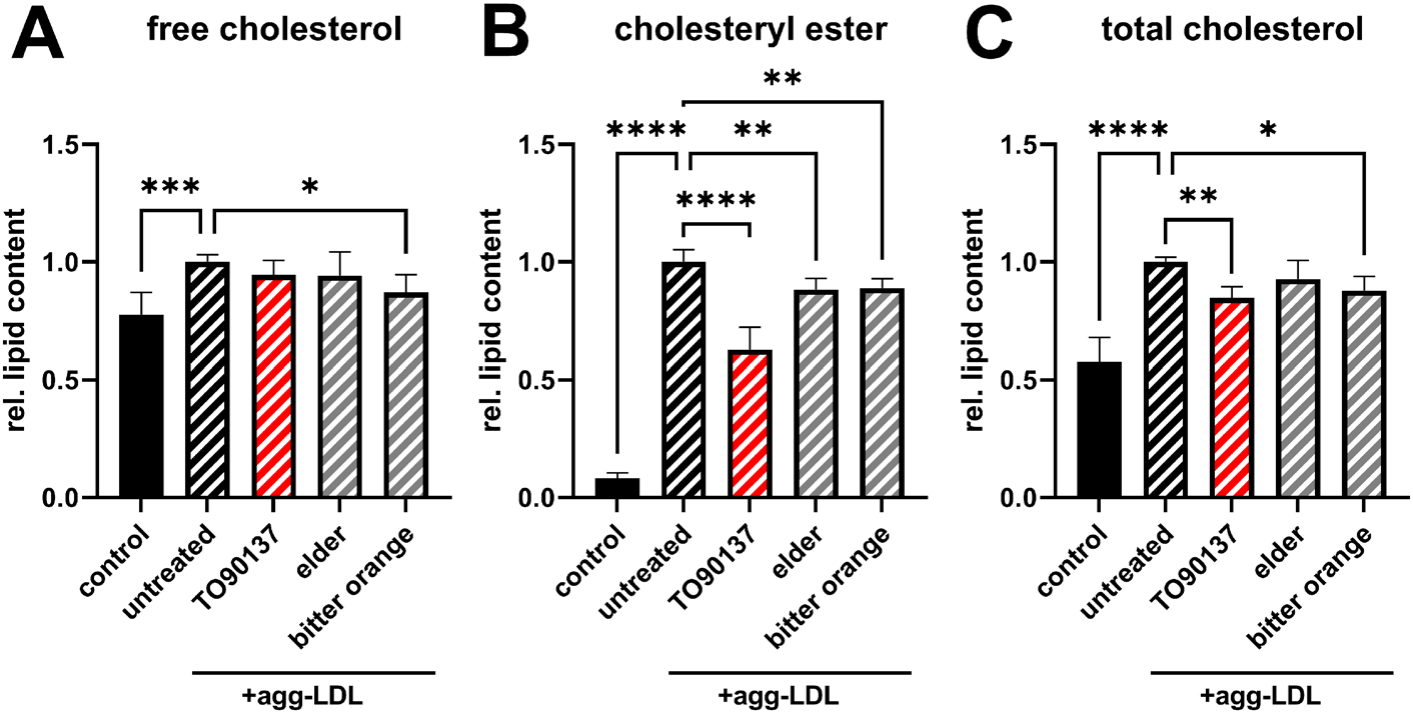
Reduction in cellular cholesterol content of foam cells by aqueous extracts of elder and bitter orange. Foam cell formation was induced in RAW264.7 by addition of aggregate LDL (agg-LDL; 200 µg/mL) for 24 h; in parallel, cells were treated with plant extracts (5–10 µg/mL) or TO901317 (10 µM). Lipids were extracted by Folch extraction and subsequently free cholesterol (**A**), cholesteryl esters (**B**) and total cholesterol levels (**C**) were measured by gas chromatography. Data represent three independent experiments performed in duplicates.

Extracts increasing ABCA1 expression were functionally validated at first priority in terms of their capability to reduce macrophage foam cell formation. Foam cell formation was induced in RAW264.7 macrophages utilizing aggregated LDL, a pathophysiologically relevant driver of atherogenesis^1^. Treatment with aggregated LDL increased cellular cholesterol levels, especially cellular cholesteryl ester levels ~ twelve-fold (Fig. 4A–C). The latter represent the storage form of excess cholesterol in lipid droplets and their cellular accumulation is a hallmark of foam cell formation. Activation of cholesterol efflux by the synthetic LXR agonist TO901317 reduced cholesteryl ester accumulation. Similarly, extracts from elder (*Sambucus nigra*) and bitter orange (*Citrus aurantium L.*) counteracted aggregated LDL-induced cholesteryl ester accumulation (Fig. 4B). In contrast, extracts from cubeb pepper (*Piper cubeba*), American spikenard (*Aralia racemose*) and neem (*Azadirachtum indica*), which were also inducers of ABCA1 expression, were not capable of reducing cholesteryl ester accumulation (Supplementary Fig. S1).

### In depth characterization of elder extract activity 

**Figure 5:**
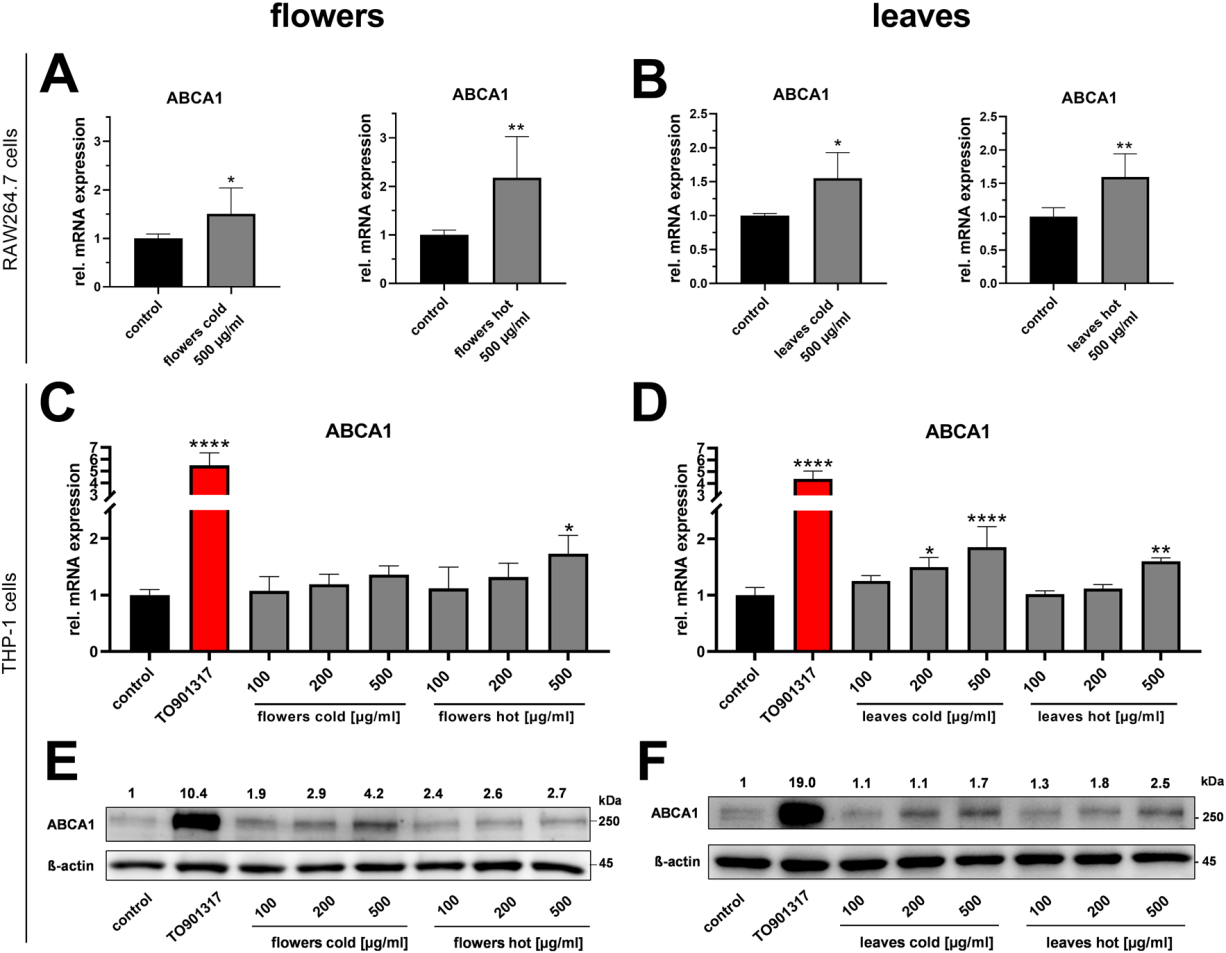
Up-regulation of ABCA1 expression by elder flower and elder leaf extracts in mouse and human macrophages. Mouse RAW264.7 macrophages (**A**, **B**) or human THP-1 monocytes differentiated towards macrophages (**C**–**F**) were treated with the indicated concentrations of cold or hot extracts of either elder flowers or elder leaves in serum-reduced medium for 24 h. TO901317 (10 µM) served as positive control. Afterwards, RNA (**A**–**D**) or proteins (**E**–**F**) were extracted and analyzed for ABCA1 expression by RT-qPCR and western blotting, respectively. Data are from experiments performed in duplicates (three to four (**A**, **B**) or two (**C**–**F**) independent experiments). (**E**, **F**) Relative protein expression is shown above blots and represent mean of data from three to four biological replicates (two independent experiments). Blots were cropped for concise presentation; original blots are shown in Supplementary Fig. S3).

Given the fact that an extract from elder (*Sambucus nigra*) was identified by our screening approach as a hitherto unknown activator of cholesterol efflux and an inhibitor of cholesteryl ester accumulation, its biological activity and composition was further investigated. Extracts prepared in-house from elder flowers and leaves by cold or hot aqueous extraction induced ABCA1 expression in RAW264.7 murine macrophages (Fig. 5A,B). In contrast, extracts from berries were inactive (data not shown).

In human THP-1 monocytes differentiated towards macrophages, extracts prepared from elder leaves or flowers induced ABCA1 mRNA expression levels dose dependently ranging from 100 to 500 µg/mL extract dry mass (Fig. 5C,D). Extracts were effective irrespectively if prepared by cold or hot aqueous extraction. Increased ABCA1 mRNA levels translated into increased ABCA1 protein expression (Fig. 5E,F), with effects that were generally even more pronounced than for mRNA expression.

**Figure 6:**
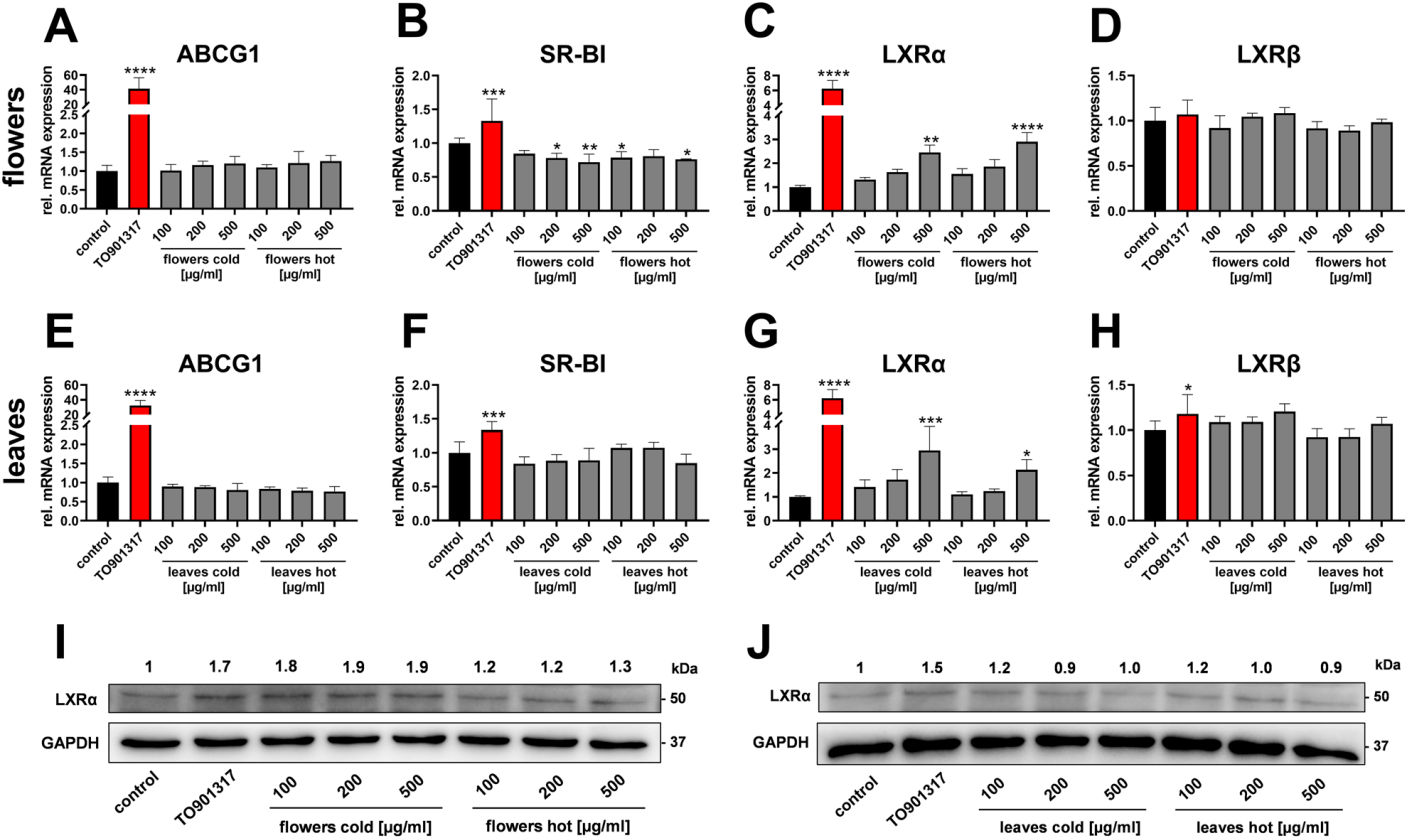
Regulation of LXR and efflux transporters by elder flower and elder leaf extracts in human macrophages. Differentiated THP-1 cells were incubated with cold or hot extracts of either elder flowers or elder leaves at the indicated concentrations in serum-reduced medium for 24 h, followed by RNA extraction. Relative mRNA expression of ABCG1 (**A**, **E**), SR-BI (**B**, **F**), LXRα (**C**, **G**) and LXRβ (**D**, **H**) was analyzed by RT-qPCR. Data from two independent experiments performed in duplicates are shown. Protein expression was analyzed by western blot (**I**, **J**). Relative protein expression is shown above blots and represent mean of data from two biological replicates, each derived from two independent experiments. Blots were cropped for concise presentation; original blots are shown in Supplementary Fig. S4.

Induction of ABCA1 expression by elder extracts was not accompanied by increased ABCG1 or SR-BI expression (Fig. 6A–D). In contrast, the expression of SR-BI was slightly down-regulated by elder flower extracts (Fig. 6C). Next, the expression of LXRs was investigated in response to elder flower and leaf extracts. These nuclear receptors respond to excess cellular cholesterol levels by enhancing cholesterol efflux via transcription of ABC-transporters including ABCA1. Expression of LXRα was induced dose-dependently by all extracts tested, while the expression of LXRβ remained unchanged (Fig. 6E–H). Changes in LXRα mRNA translated into increased protein expression levels for elder flower extracts, while the effect was less pronounced for leaf extracts (Fig. 6I,J).

In summary, extracts from elder leaves as well as flowers inhibit cholesteryl ester accumulation in macrophages, which is likely mediated by increased transcription of ABCA1 via LXRs.

### Chlorogenic acid and rutin are the main polyphenols in elder extracts

Given the fact that elder is rich in polyphenols^17^, a panel of polyphenols were analyzed in extracts from elder flowers and leaves by HPLC and identified and quantitated using authentic standards (Table 1 and Supplementary Fig. S2). Remarkably, a comparable pattern of polyphenols was observed in all extracts, regardless if prepared from flowers or leaves. Chlorogenic acid and its derivatives as well as rutin (Quercetin-3-O-glucorhamnosid) were present in all extracts tested, whereas isoquercetin was present in flower extracts only. Chlorogenic acid was most abundant in leaf extracts (28.6–31.9 mg/g dry matter). Rutin concentrations were highest in leaf and flower extracts prepared by hot aqueous extraction (23.5–28.2 mg/g dry extract).

**Table 1:** Contents of phenolic compounds identified by HPLC analysis in elder flower and elder leaf extracts.

Compound name		Content							
		Elder leaves cold		Elder leaves hot		Elder flowers cold		Elder flowers hot	
		mg/mL extract	mg/g dry matter	mg/mL extract	mg/g dry matter	mg/mL extract	mg/g dry matter	mg/mL extract	mg/g dry matter
**1**	Neochlorogenic acid (5-CQA)	0.025	0.923	0.022	0.655	0.023	0.431	0.019	1.038
**2**	Chlorogenic acid (3-CQA)	0.865	31.926	0.961	28.586	0.176	3.377	0.139	7.484
**3**	Chlorogenic acid isomer (4-CQA)	0.017	0.620	0.019	0.566	0.019	0.366	0.012	0.640
**4**	Rutin	0.319	11.775	0.948	28.214	0.678	12.990	0.436	23.452
**5**	Isoquercetin	n.d.^(^^a)^	n.d	n.d	n.d	0.029	0.548	0.017	0.903

^(a)^n.d.: beyond quantitation limit.

Bold numbers correspond to peaks as indicated in the chromatograms depicted in Supplementary Fig. S2.

### Elder extracts do not induce hepatic lipogenic gene expression

**Figure 7:**
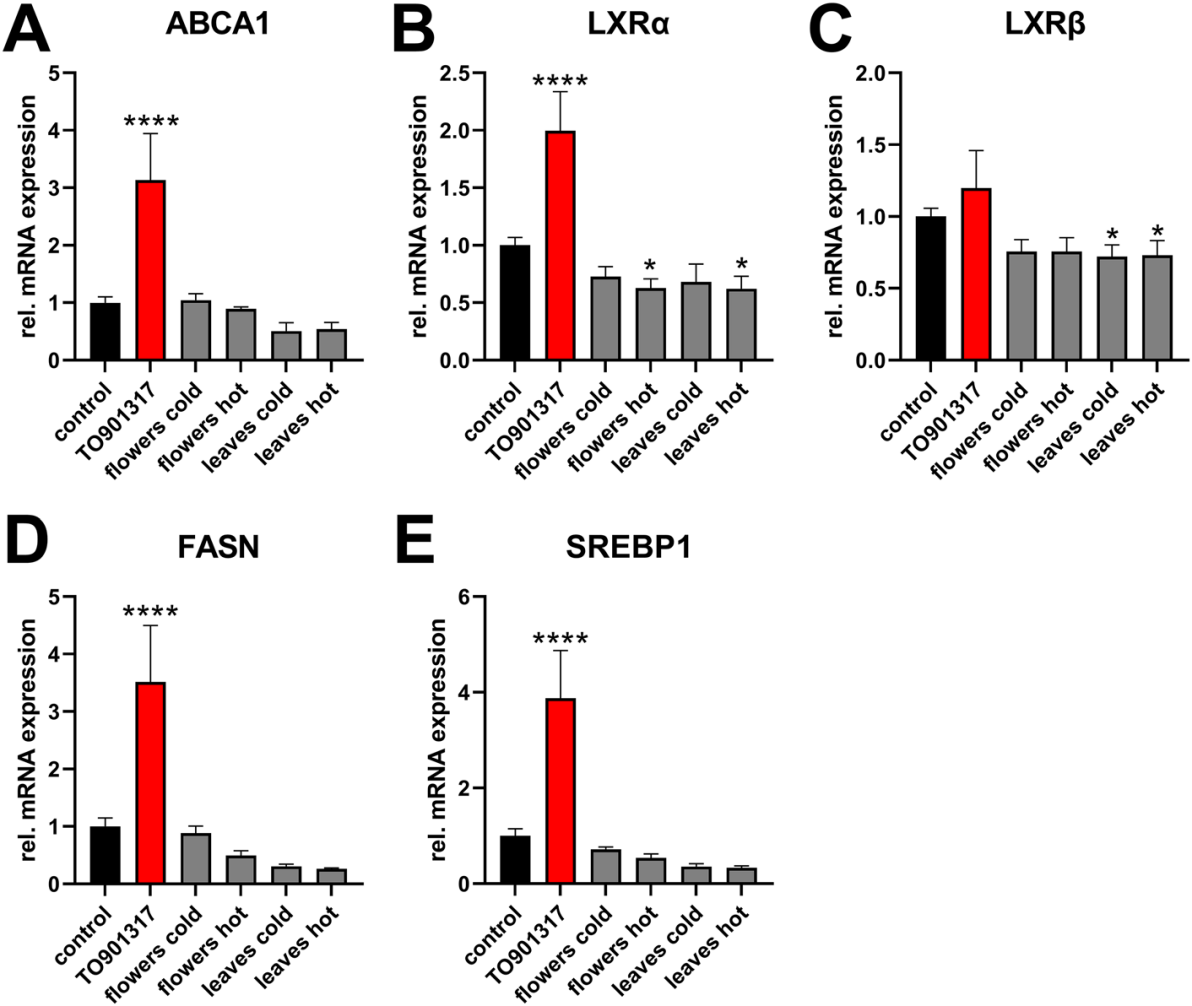
Lipogenic genes are not induced by elder extracts in hepatic cells. Huh-7 cells were treated for 24 h with cold or hot extracts of either elder flowers or elder leaves (500 µg/mL), followed by RNA extraction. Relative mRNA expression of ABCA1 (**A**) LXRα (**B**), LXRβ (**C**), and FASN (**D**) and SREBP1 (**E**) was analyzed by RT-qPCR. Data from two independent experiments performed in duplicates are shown.

Pharmacological activation of LXR in humans beneficially affects cholesterol efflux and HDL biogenesis, but also triggers hepatic lipogenesis. Therefore, potential effects of elder extracts on the gene expression levels of ABCA1 and LXRs as well as lipogenic genes were investigated in human Huh-7 hepatocyte-derived cells. Unlike TO901317, no induction of LXRα or ABCA1 expression was observed for elder extracts in Huh-7 cells (Fig. 7A–C).

As expected, LXR activation by its synthetic agonist TO901317 induced the expression of fatty acid synthase (FASN) and its transcriptional regulator sterol-response element-binding protein 1 (SREBP1), indicative of induced lipogenesis (Fig. 7D,E). These adverse effects were not observed by any of the elder extracts tested. Altogether these data indicate that elder extracts induce ABCA1 in a macrophage specific manner without adversely affecting hepatic lipogenic gene expression.

## Discussion

Here we present a screening and validation strategy to identify natural extracts that increase macrophage cholesterol efflux with the overall aim to characterize novel agents that counteract foam cell formation in atherosclerosis. Via screening and incremental validation in human and mouse macrophage models, extracts from elder flowers and leaves were identified to increase cholesterol efflux. Additionally, elder extracts partially reversed aggregated LDL-induced cholesteryl ester accumulation and induced expression of ABCA1, putatively by the induction of LXR.

For screening purpose, an assay measuring the efflux of fluorescent BODIPY-cholesterol (also termed TopFluor-cholesterol) was established. While the gold-standard for quantifying cholesterol efflux is based on radiolabeled cholesterol, such methodologies are unsuitable for screening assays. BODIPY-cholesterol mimics the behavior of cholesterol in cell membranes^18^ as well as its cellular localization pattern^19^. On the other hand, partitioning into lipid droplets is higher for BODIPY-cholesterol than for intrinsic sterols^20^ and relative cholesterol efflux rates are higher than for radiolabeled cholesterol^13^. Despite this, excellent correlation between efflux rates of radiolabeled cholesterol and BODIPY-cholesterol were observed^13^ and our data indicate that efflux of the latter is responsive to LXR-activation (compare Fig. 1B). Most importantly, top hits were confirmed as inducers of cholesterol efflux using radiolabeled cholesterol, thus substantiating the usefulness of the applied fluorescence-based assay utilizing Bodipy-cholesterol.

Further evidence for the validity of the newly developed screening assay comes from the re-identification of bitter orange (*Citrus aurantium L.*): Others have shown that isolated flavonoids of bitter orange reduce foam cell formation and increase ABCA1 expression in RAW264.7 cells^21^ More importantly, citrus flavonoids suppress atherogenesis in vivo in murine models of atherosclerosis^22^. In contrast, the identification of elder as an inhibitor of foam cell formation is, to the best of our knowledge, novel.

Crucial events modulating the development of atherosclerotic plaques include the uncontrolled uptake of LDL and aggregated LDL—rather than oxidized LDL^1^—into tissue resident macrophages and subsequent foam cell formation. This process can be partially antagonized by HDL-mediated cholesterol flux from the periphery towards the liver and excretion via bile, which is termed RCT. The LXR-ABCA1-axis is a significant positive regulator of this process. LXR is the major transcription regulator of ABCA1 (amongst other ABC transporters such as ABCG1). Activation of macrophage LXR by excess sterols followed by cholesterol efflux via ABCA1 is regarded protective against foam cell formation and atherosclerosis^23,24^. LXR is encoded by two genes, LXRα (NR1H3) and LXRβ (NR1H2). The former is expressed in tissues such as macrophages, liver and adipose tissue, while LXRβ is ubiquitously expressed. The increase in LXRα mRNA in THP-1 macrophages by elder extracts (compare Fig. 6) is in accordance with positive autoregulation of LXRα (but not LXRβ) and thus indicates induced transcriptional activity^25^.

While it promotes cholesterol efflux in peripheral macrophage foam cells, the LXR-ABCA1 axis in the liver contributes to HDL biogenesis. Our data show that the induction of the LXR-ABCA1-axis by extracts of elder is restricted to macrophages. This might be advantageous compared to tissue-independent LXR activation. Indeed, clinical studies using LXR agonists were hindered by the development of non-alcoholic fatty liver diseases (NAFLD), since LXR is also an activator of the transcription factor SREBP1 (gene name: SREBF1) that induces hepatic lipogenesis via fatty acid synthase^26^. To date, no LXR agonist was able to be applied for clinical use due to a simultaneous activation of lipogenesis^27^. In contrast, our study reveals that elder extracts did not increase expression of SREBP1 and FASN in hepatic cells, indicating no safety concerns regarding the development of NAFLD if elder extracts are intended to be used to ameliorate atherosclerosis.

Elder’s differential effect on the regulation of the LXR-ABCA1 axis in macrophages versus hepatocytes remains to be investigated. Several mechanisms have been proposed that account for cell type specific regulation of LXR such as differential response to LXR’s endogenous ligand desmosterol^28^. In addition, non-coding RNAs have been proposed to regulate LXR activity in a tissue specific manner^29,30^.

LXR is a pleiotropic regulator of gene expression and, besides regulating cholesterol efflux and lipogenesis, also regulates inflammation^31,32^. In particular, ABCA1-dependent changes in membrane lipid organization activates the expression of anti-inflammatory genes in macrophages in response to LXR-activation^33^. As it is commonly accepted that atherosclerosis is an inflammatory disease, elder leaves and flowers may contribute to atheroprotection not only by the reduction of foam cell formation, but also through reduction of inflammation. Interestingly, elder leaves in fact possess anti-inflammatory activity in human neutrophils^34^. Further, elder was shown to improve HDL function and ameliorate atherosclerosis in mice^35,36^. However, the later effects are restricted to elder berries, which were ineffective in inducing ABCA1 expression in our study.

Our findings of abundant content of rutin, a quercetin glycoside, as well as of chlorogenic acid in elder flowers is in accordance with literature^37,38^. Analysis of extracts from elder leaves revealed likewise a considerable content of the aforementioned polyphenols. Most interestingly, chlorogenic acid induced macrophage cholesterol efflux, reduced foam cell formation and protected against atherosclerosis in mice^39^. In addition, rutin reduced macrophage foam cell formation in vitro^40^. We therefore hypothesize that chlorogenic acid and rutin are potential candidates for the biologically active compounds in elder extracts regarding the regulation of ABCA1 and cholesterol efflux. Of note, further studies are required for substantiation.

In conclusion, the increase in ABCA1 expression and cholesterol efflux and the reduction in foam cell formation make elder extracts a putative anti-atherosclerotic food supplement that warrants its further testing in in vivo settings. The absence of such in vivo evidence is—despite the use of two independent macrophage cell models—a limitation of our study. While elder flowers are widely used to prepare syrup in Central Europe and beyond, elder leaves are not traditionally used in nutrition. Therefore, the usage of elder flowers in nutritional supplements is a straightforward approach, while the usage of leaves might require further testing, for instance for potential toxicity.

In summary, targeting macrophage cholesterol efflux by elder extracts could be of particular benefit in addition to lowering LDL cholesterol for instance by lifestyle modification, food supplements or medication and is regarded as safe in terms of hepatic side effects.

### Supplementary Information

Supplementary Information.

## Data Availability

The data that support the findings of this study are available from the corresponding author, C.R., upon reasonable request.
