# Supplementary material for: Elder (Sambucus nigra), identified by high-content screening, counteracts foam cell formation without promoting hepatic lipogenesis
Source: Sci Rep. 2024 Feb 12;14:3547. doi: 10.1038/s41598-024-54108-7 (PMC10861454; doi:10.1038/s41598-024-54108-7)

Supplementary Information to

Elder (*Sambucus nigra*), identified by high-content screening, counteracts foam cell formation without promoting hepatic lipogenesis

Stefanie Steinbauer, Alice König, Cathrina Neuhauser, Bettina Schwarzinger, Herbert Stangl, Marcus Iken, Julian Weghuber and Clemens Röhrl

Table S1. List of primers used in RT-qPCR experiments.

gene	species	forward primer (5'-3')	reverse primer (5'-3')	accession number	annealing temp
ABCA1	*Mus musculus*	CTTGTTGGCCTCAGTTAAG	GACGGCATGGCTTTATTC	NM_013454.3	57 °C
ABCG1	*Mus musculus*	CTTCCAAGTGGTGTCTCT	ACCCAGATCCCTCAGATA	NM_009593.2	57 °C
SCARB1	*Mus musculus*	CTATGACGATCCCTTCGT	CTGAGTCCGTTCCATTTG	NM_016741.2	57 °C
HPRT1	*Mus musculus*	CCCTGGTTAAGCAGTACA	CAAGGGCATATCCAACAAC	NM_013556	57 °C
GUSB	*Mus musculus*	TTCGTACCAGCCACTATC	CACAGACCACATCACAAC	NM_010368	57 °C
ABCA1	*Homo sapiens*	GAGGATGTCCAGTCCAGTAA	CACAATACCAGCCCAGAAC	NM_005502.4	60 °C
ABCG1	*Homo sapiens*	GGACCTTTCCTATTCGGTTC	GTTCATCAGCGTGGACTT	NM_016818.3	60 °C
SCARB1 (SR-BI)	*Homo sapiens*	GTCCTCGCTGGAGTTCTA	GGTGCTGACGTTCTGAAT	NM_001367987.1	60 °C
NR1H3 (LXRα)	*Homo sapiens*	GAGACATCTCGGAGGTACAA	GCAATGAGCAAGGCAAAC	NM_001363595.2	60 °C
NR1H2 (LXRβ)	*Homo sapiens*	ACAACCACGAGACAGAGT	GGCCGAGAAGATGTTGATG	NM_001256647.3	60 °C
RPL5	*Homo sapiens*	TGGGCCAGAATGTTGCAGAT	AGGGACATTTTGGGACGGTT	NM_000969	60 °C
GAPDH	*Homo sapiens*	TGGTATCGTGGAAGGACTCA	CAGTGAGCTTCCCGTTCAG	NM_002046	60 °C
B2M	*Homo sapiens*	TGAAGCTGACAGCATTCG	CAGACACATAGCAATTCAGG	NM_004048	60 °C
FASN	*Homo sapiens*	CCATCTACAACATCGACACC	GCACCACATCCTCAAACA	NM_004104.5	60 °C
SREBF1 (SREBP1)	*Homo sapiens*	GGTCGTAGATGCGGAGAA	GCGCAAGACAGCAGATTTA	NM_001388394.1	60 °C

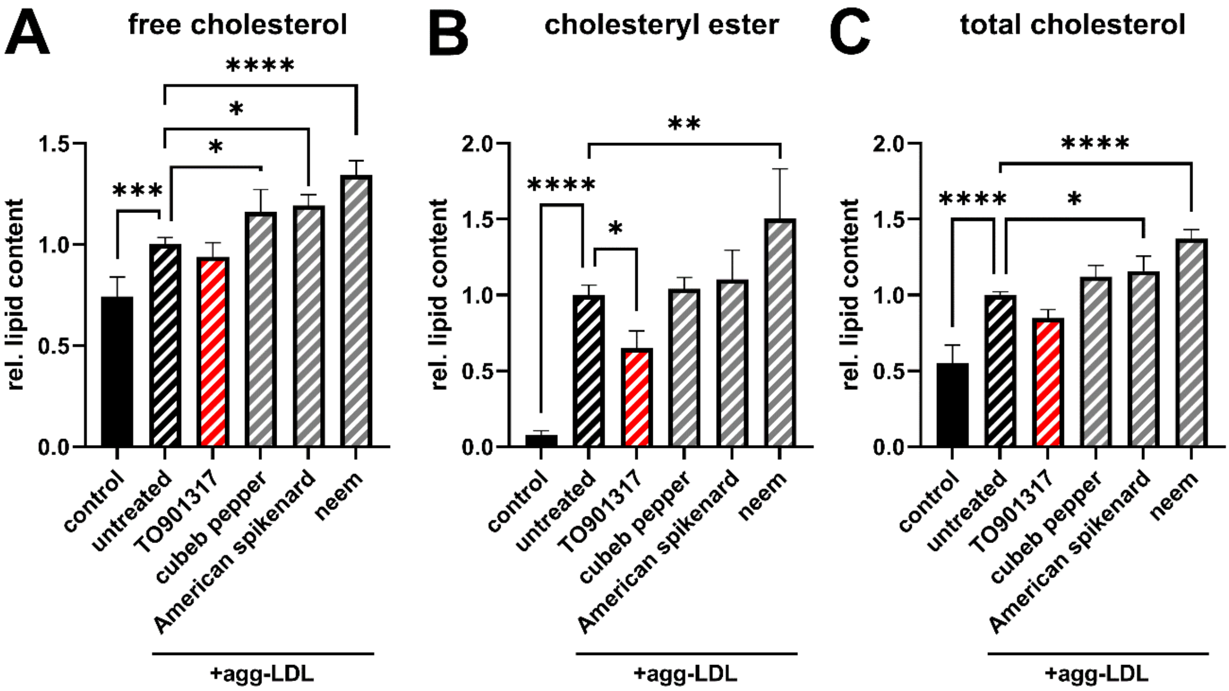

Figure S1: No beneficial effect of aqueous extracts of cubeb pepper, American spikenard and neem on cellular cholesterol content of RAW264.7 foam cells. Foam cell formation was induced by the addition of aggregated LDL (agg-LDL; 200 µg/ml) for 24 h; in parallel, cells were treated with plant extracts (PECKISH library, ~5-10 µg/ml) or TO901317 (10 µM). Lipids were extracted by Folch extraction and subsequently free cholesterol (A) and cholesterol ester (B) were analyzed by gas chromatography. Total cholesterol (C) was calculated as the sum of free and esterified cholesterol. Data were normalized to cellular protein content and are expressed relative to values of cells treated with agg-LDL only. Two independent experiments performed in duplicates are shown and statistically significant differences are indicated as * ($p \leq 0.05$), ** ($p \leq 0.01$), *** ($p \leq 0.001$) or **** ($p \leq 0.001$).

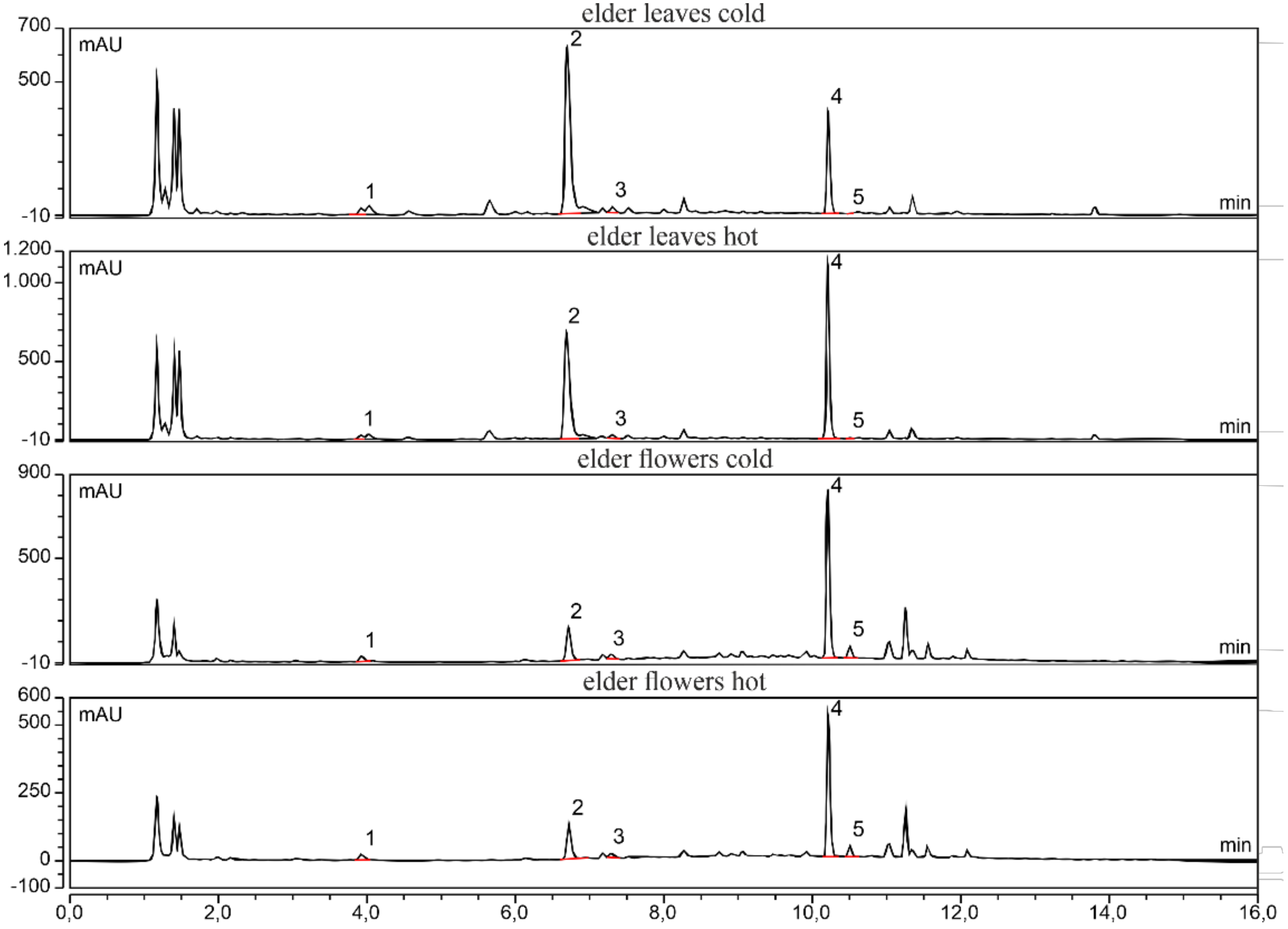

Figure S2: Chromatograms of HPLC analyses of elder extracts. Neochlorogenic acid (5-CQA; peak #1); chlorogenic acid (3-CQA; peak #2); chlorogenic acid isomer (4-CQA; peak #3), rutin (peak #4) and and isoquercetin (peak #5) were identified. Quantitation is given in Table 1 in the main manuscript.

Figure S3: Original blots for cropped blots presented in figure 5 (main manuscript). Blots were cut prior to hybridisation with antibodies.

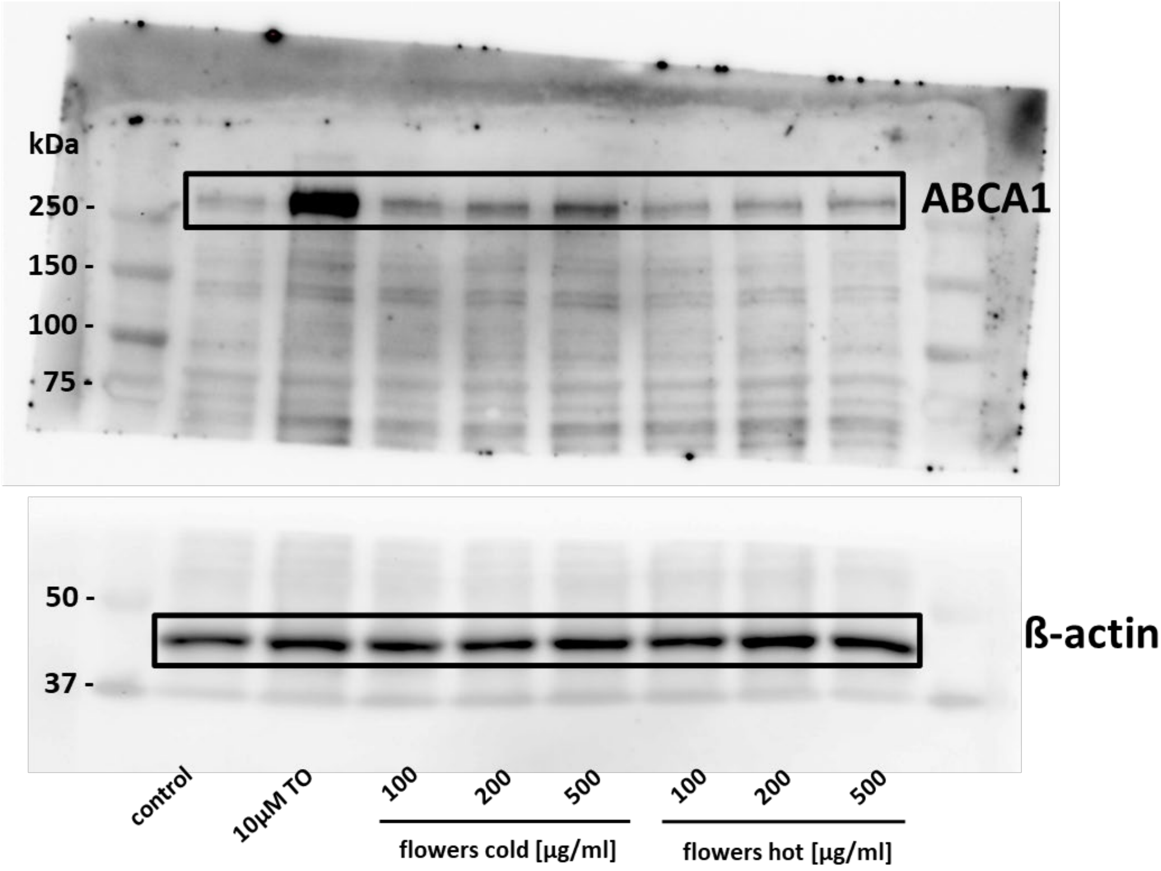

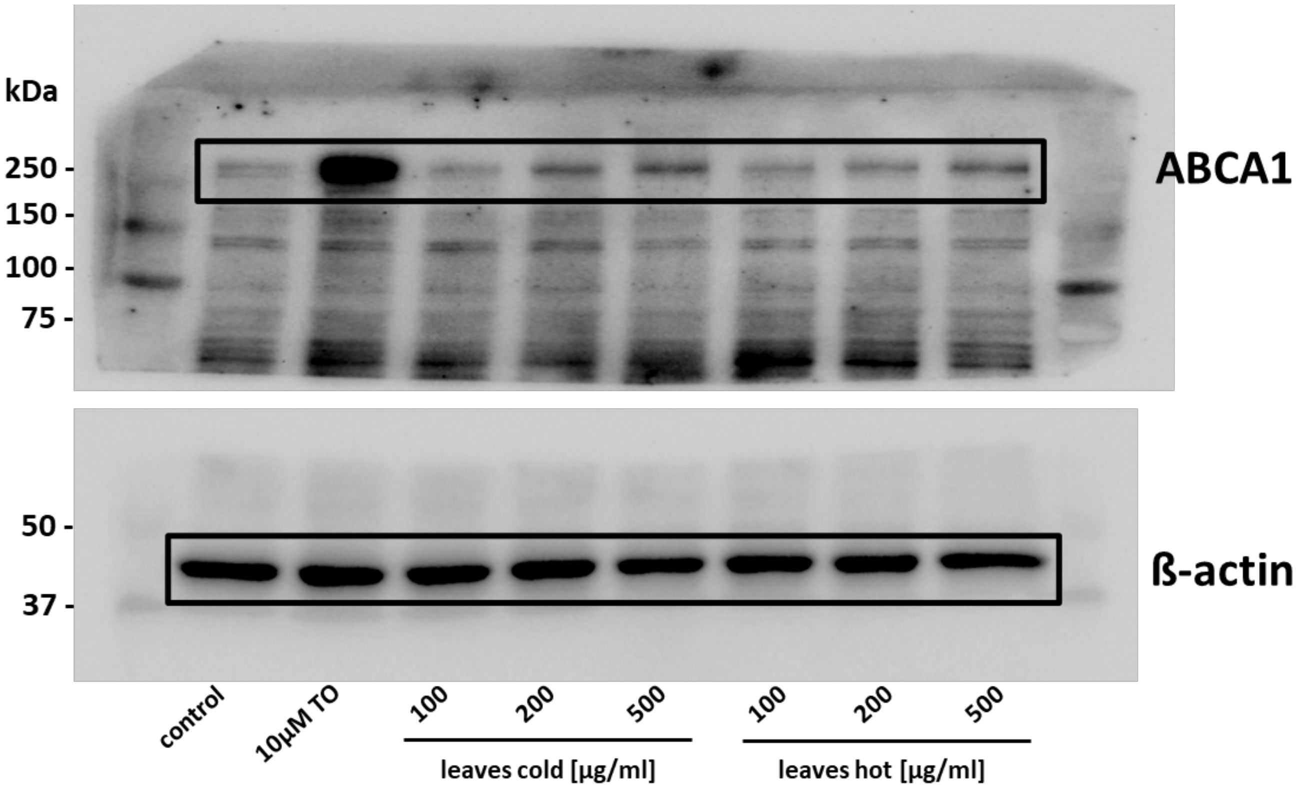

Figure S4: Original blots for cropped blots presented in figure 6 (main manuscript). Blots cut prior to hybridisation with antibodies.

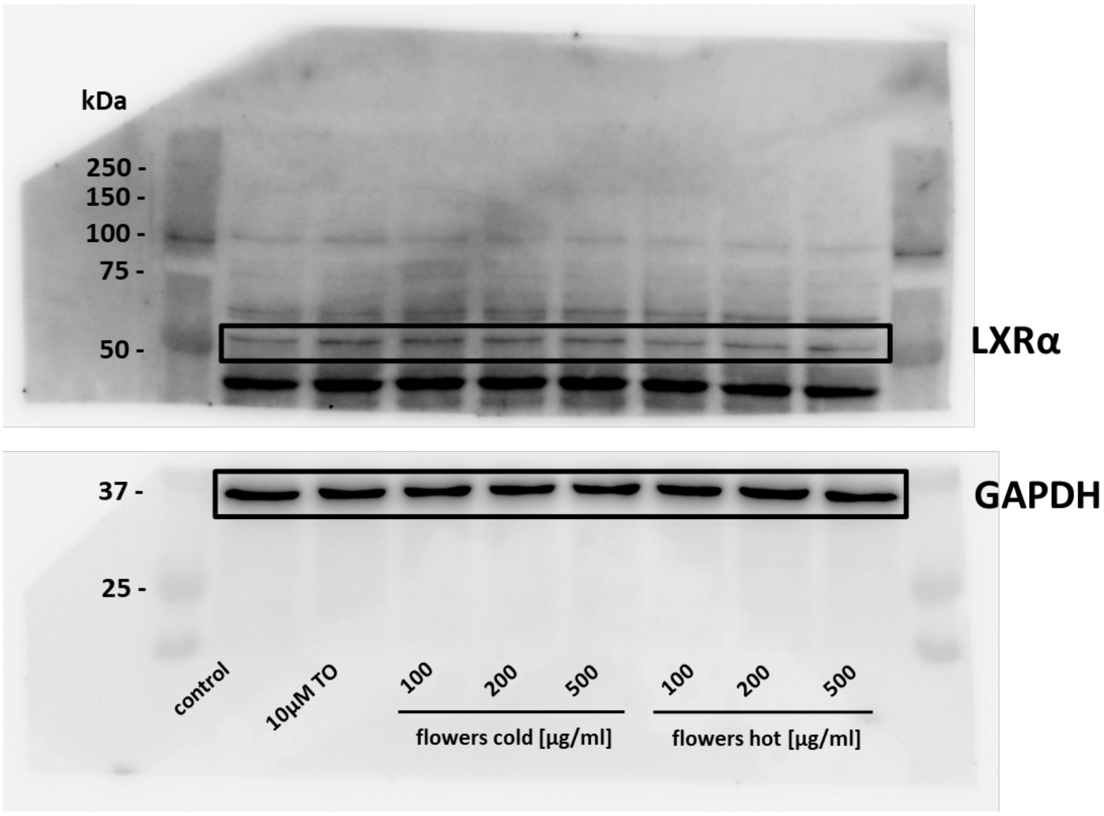

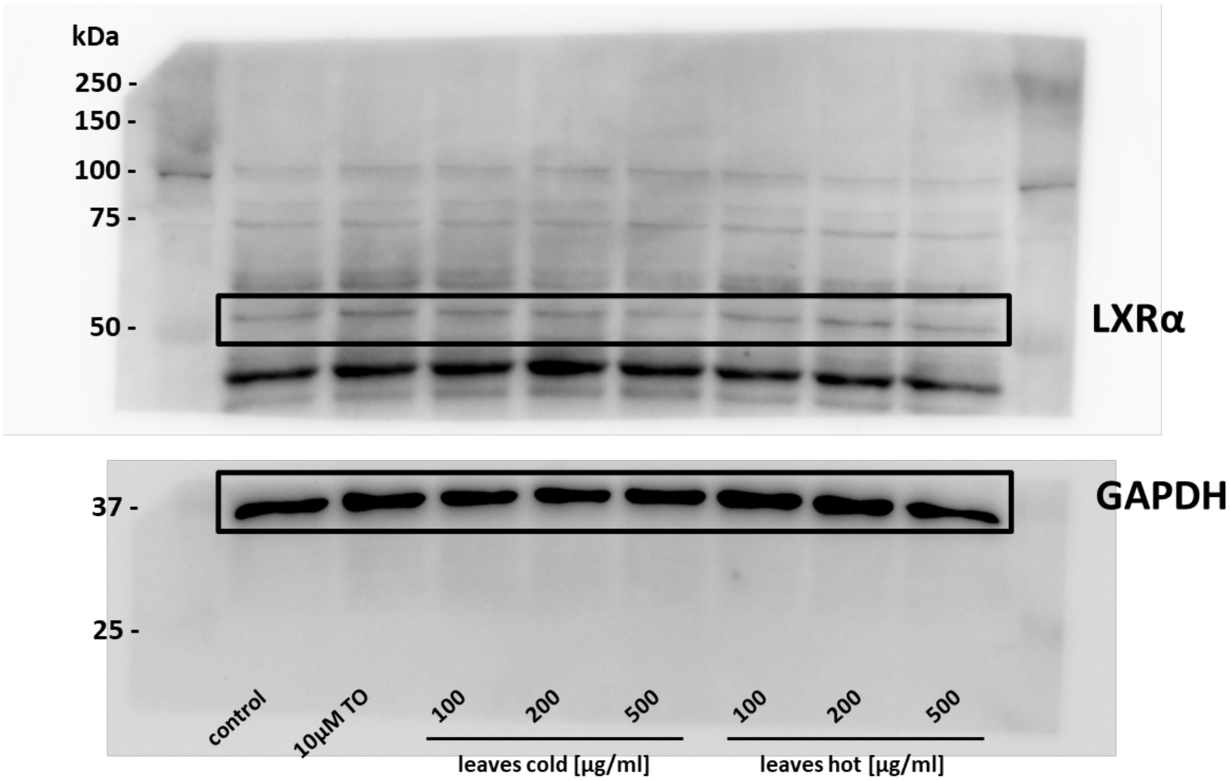

Supplement: Supplementary file 1 — Supplementary Information. [file 41598_2024_54108_MOESM1_ESM.pdf]
